# Expression of NGF/proNGF and Their Receptors TrkA, p75^NTR^ and Sortilin in Melanoma

**DOI:** 10.3390/ijms23084260

**Published:** 2022-04-12

**Authors:** Mark Marsland, Amiee Dowdell, Chen Chen Jiang, James S. Wilmott, Richard A. Scolyer, Xu Dong Zhang, Hubert Hondermarck, Sam Faulkner

**Affiliations:** 1 School of Biomedical Sciences and Pharmacy, College of Health, Medicine and Wellbeing, University of Newcastle, Callaghan, NSW 2308, Australia; mark.marsland@uon.edu.au (M.M.); amiee.dowdell@uon.edu.au (A.D.); xu.zhang@newcastle.edu.au (X.D.Z.); sam.faulkner@newcastle.edu.au (S.F.); 2 Hunter Medical Research Institute, University of Newcastle, New Lambton Heights, NSW 2305, Australia; chenchen.jiang@newcastle.edu.au; 3 School of Medicine and Public Health, College of Health, Medicine and Wellbeing, University of Newcastle, Callaghan, NSW 2308, Australia; 4 Tissue Pathology and Diagnostic Oncology, Royal Prince Alfred Hospital and NSW Health Pathology, Sydney, NSW 2050, Australia; james.wilmott@melanoma.org.au (J.S.W.); richard.scolyer@sswahs.nsw.gov.au (R.A.S.); 5 Melanoma Institute Australia, The University of Sydney, Sydney, NSW 2065, Australia; 6 Faculty of Medicine and Health, The University of Sydney, Sydney, NSW 2050, Australia; 7 Charles Perkins Centre, The University of Sydney, Sydney, NSW 2006, Australia

**Keywords:** melanoma, NGF, proNGF, TrkA, p75^NTR^, sortilin

## Abstract

There is increasing evidence that nerve growth factor (NGF) and its receptors, the neurotrophic receptor tyrosine kinase 1 (NTRK1/TrkA), the common neurotrophin receptor (NGFR/p75^NTR^) and the membrane receptor sortilin, participate in cancer growth. In melanoma, there have been some reports suggesting that NGF, TrkA and p75^NTR^ are dysregulated, but the expression of the NGF precursor (proNGF) and its membrane receptor sortilin is unknown. In this study, we investigated the expression of NGF, proNGF, TrkA, p75^NTR^ and sortilin by immunohistochemistry in a series of human tissue samples (*n* = 100), including non-cancerous nevi (*n* = 20), primary melanomas (*n* = 40), lymph node metastases (*n* = 20) and distant metastases (*n* = 20). Immunostaining was digitally quantified and revealed NGF and proNGF were expressed in all nevi and primary melanomas, and that the level of expression decreased from primary tumors to melanoma metastases (*p* = 0.0179 and *p* < 0.0001, respectively). Interestingly, TrkA protein expression was high in nevi and thin primary tumors but was strongly downregulated in thick primary tumors (*p* < 0.0001) and metastases (*p* < 0.0001). While p75^NTR^ and sortilin were both expressed in most nevi and melanomas, there was no significant difference in expression between them. Together, these results pointed to a downregulation of NGF/ProNGF and TrkA in melanoma, and thus did not provide evidence to support the use of anti-proNGF/NGF or anti-TrkA therapies in advanced and metastatic forms of melanoma.

## 1. Introduction

Melanoma is an aggressive malignancy emerging from neural crest-derived melanocytes. Melanoma is often fatal due to resistance to therapy and aggressive metastases throughout the body [1]. Melanoma incidence is increasing and the identification of signaling pathways that lead to melanoma cell invasion and metastasis would provide novel therapeutic strategies to limit disease progression.

During development, melanocytes emerge from neural crest-derived cells, which have migrated throughout the epidermis [2]. A neural crest is a multipotent and highly migratory embryonic cell population, and melanocytes have the potential to transform into aggressive melanoma. Neural crest cell migration, proliferation and differentiation into melanocytes is influenced by many growth factors, including those involved in neurogenesis. Nerve growth factor (NGF) has been shown to enhance melanocyte precursor cell proliferation and migration through the activation of the tropomyosin-related kinase A (TrkA) membrane receptor and the common neurotrophin receptor p75^NTR^ (also called CD271) [3]. Whereas TrkA is a tyrosine kinase receptor that activates multiple intracellular signaling cascades, such as the mitogen-activated protein kinases (MAPK) or the phospholipase C (PLC) signaling pathway, p75^NTR^ is a cell death/survival receptor and a member of the tumor necrosis factor receptors [4]. In the adult epidermis, NGF has a paracrine effect in stimulating the survival and proliferation of both epidermal cells and melanocytes [5]. NGF stimulation of TrkA and p75^NTR^ also enhances proliferation and migration of melanoma cells [3]. P75^NTR^ appears to be essential in tumor initiation, phenotype switching and reprogramming of metastatic melanoma [6,7,8] and TrkA has been reported to activate cell proliferation [9]. However, there are contradictory findings reporting that TrkA induces an anti-proliferative response in melanoma cells [10]. Interestingly, in clinical trials the tyrosine kinase inhibitor entrectinib can inhibit the growth of melanoma tumors [11], especially in spitzoid melanoma [12] where there is higher occurrence of NTRK fusion (21–29%) compared to cutaneous melanoma (<1%) [13]. As entrectinib also inhibits other Trk receptors, as well as ROS1 and ALK, it is unclear if the therapeutic effectiveness of entrectinib is due to targeting TrkA or other sensitive receptors. In addition, the precursor for NGF (proNGF) can also regulate cell growth [4], but it is unknown if proNGF and its membrane receptor sortilin are also involved in melanoma. Together, it appears that the data about the expression of NGF, proNGF and their receptors in melanoma are incomplete and fragmentary.

In the present study we aimed to clarify the protein expression of NGF, proNGF and their receptors TrkA, p75^NTR^ and sortilin in melanoma by using immunohistochemistry. Our data showed that p75^NTR^ and sortilin were expressed in most primary tumors and metastases, whereas NGF/proNGF and TrkA were downregulated in primary melanoma and metastases. In particular, TrkA was strongly downregulated in melanoma and therefore the literature supporting the effectiveness of entrectinib in melanoma might point to the inhibition of other pathways rather than those activated by TrkA.

## 2. Results

### 2.1. NGF Expression in Melanoma

**Figure 1:**
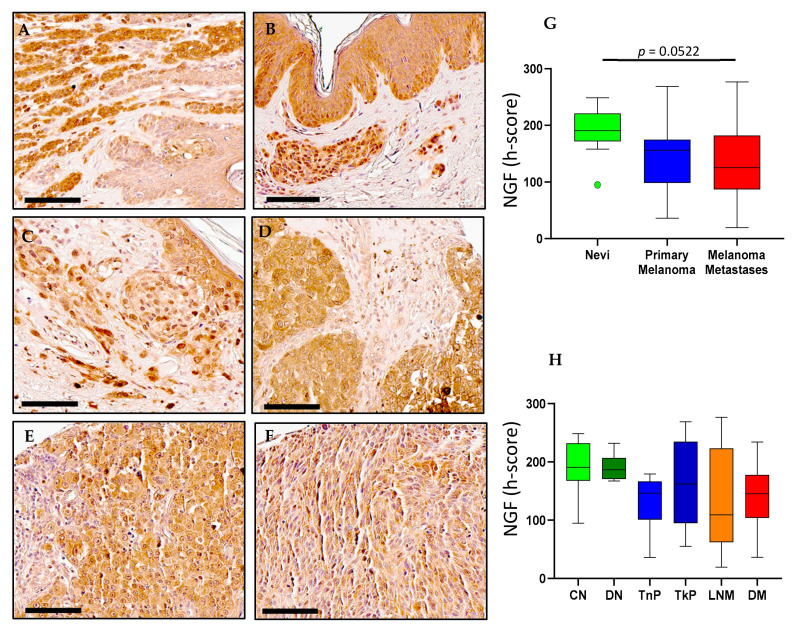
**NGF expression in nevi**, **primary melanoma and metastatic human tissues** (**A**–**F**). (**A**) Immunohistochemical detection of NGF, representative pictures are shown for compound nevi, (**B**) dysplastic nevi, (**C**) thin primary melanomas, (**D**) thick primary melanomas, (**E**) lymph node metastases and (**F**) distant metastases. (**G**) Digital quantification of NGF staining intensities according to grouped pathological subtypes: nevi (h-score = 190.7, IQR 171.1–248.7), primary melanomas (h-score = 155.9, IQR 98.46–174.8) and metastases (h-score = 125.3, IQR 86.88–182.2). (**H**) NGF staining intensities for individual pathological subtypes: CN = compound nevi (h-score = 190.7, IQR 167.5–232.1), DN = dysplastic nevi (h-score = 186.7, IQR 170.6–206.9), TnP = thin primary (h-score = 146.4, IQR 100.8–166.7), TkP = thick primary (h-score = 162.2, IQR 94.89–234.8), LNM = lymph node metastasis (h-score = 109.1, IQR 62.23–276.7), DM = distant metastasis (h-score = 145.3, IQR 103.9–177.8). Scale bar = 90 µm. Data are expressed as medians (horizontal line in the center of the box) and box limits indicate the interquartile range (IQR) with the whiskers extending 1.5 times the IQR from the 25th and 75th percentiles; outliers are represented by dots. H-score distributions were compared using the Wilcoxon rank-sum (dichotomous) or Kruskal–Wallis (multiple comparisons) tests (*p* < 0.05).

**Figure 2:**
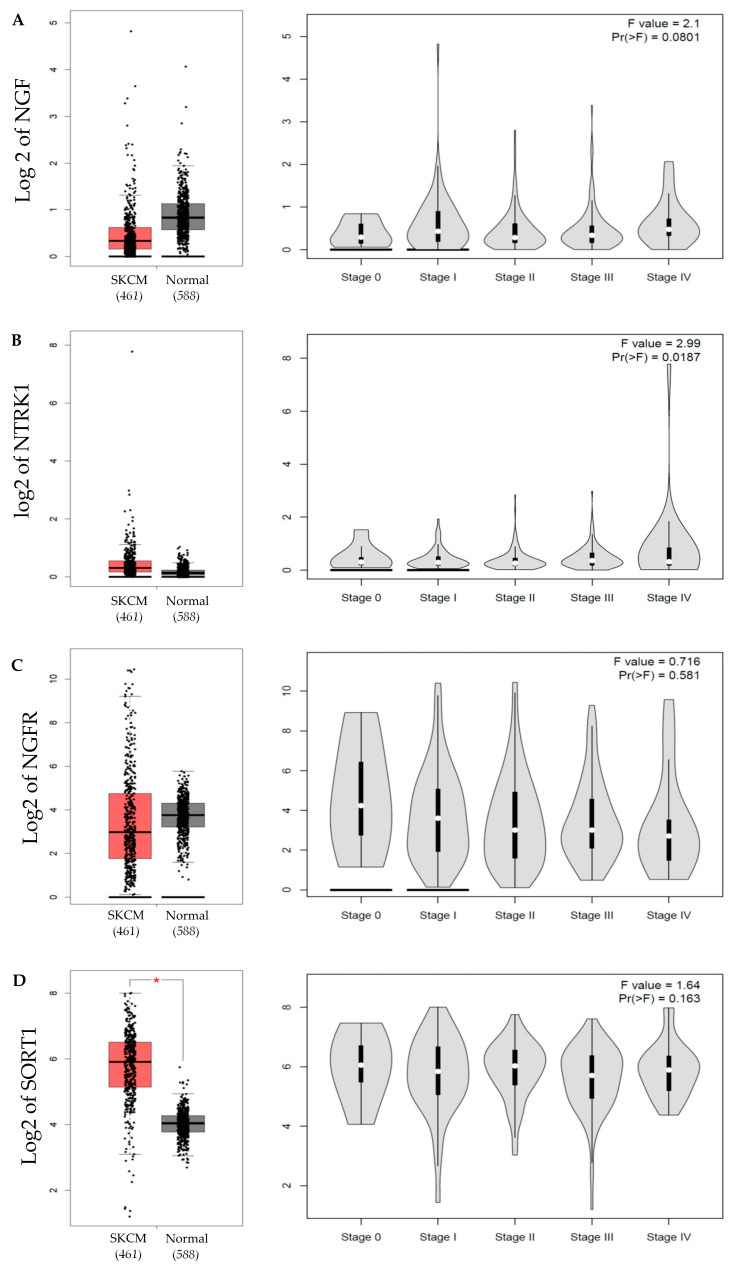
**Transcription expression of NGF** (**A**), **TrkA** (**B**), **p75^NTR^** (**C**) **and sortilin** (**D**). (**Left**) skin cutaneous melanoma (SKCM) vs. normal skin tissue (normal). Sortilin mRNA expression was significantly higher in melanoma than in normal skin (* *p* < 0.01). (**Right**) Transcriptional levels of NGF, TrkA, p75^NTR^ and sortilin across melanoma stages 1–5. The method for differential analysis of gene expression was one-way ANOVA, using disease state (SKCM or normal) as variables for box plots and pathological stages (stage 0–5) as variables for stage plots. For normal skin, GEPIA sourced the GTEx project (https://gtexportal.org/home/) (accessed on 20 July 2021).

**Figure 3:**
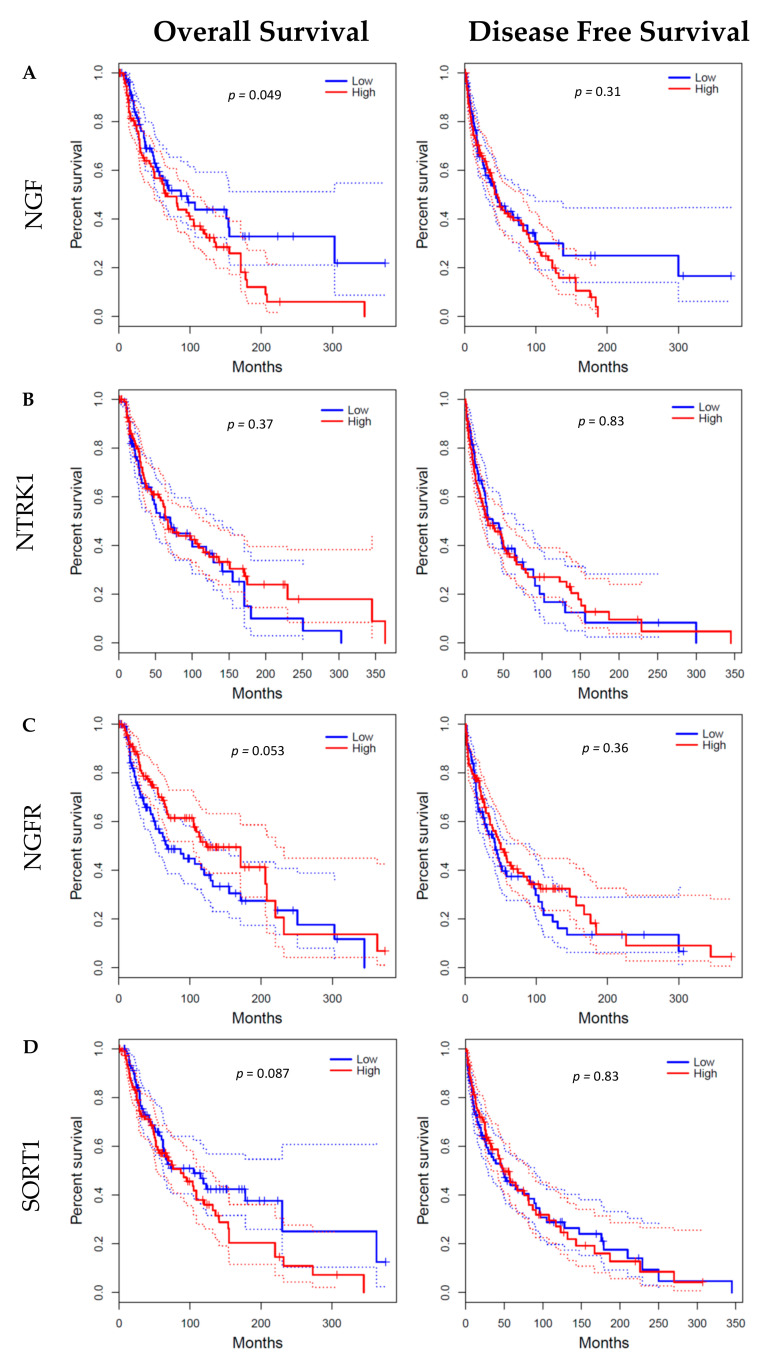
The overall survival (OS) and disease-free survival (DFS) analysis for NGF (**A**), TrkA (**B**), p75^NTR^ (**C**) and sortilin (**D**) gene expression in melanoma determined by the GEPIA database. OS (**left**) and DFS (**right**) analyses based on low and high expression of (**A**) NGF, (**B**) TrkA, (**C**) p75^NTR^ and (**D**) sortilin in melanoma (GEPIA from TCGA). High NGF expression was associated with shorter overall survival (*p* = 0.049 log-rank test). High (red) cut-off = 75%, low (blue) cut-off = 25%.

NGF immunohistochemical staining was observed in all cases of compound nevi (Figure 1A), dysplastic nevi (Figure 1B), thin primary melanomas (Figure 1C), thick primary melanomas (Figure 1D), lymph node metastases (Figure 1E) and distant metastases (Figure 1F). Digital quantification revealed no statistical differences (*p* = 0.0522) between the grouped pathological subtypes of nevi (h-score = 190.7, IQR 171.1–248.7), primary melanomas (h-score = 155.9, IQR 98.46–174.8) and metastases (h-score = 125.3, IQR 86.88–182.2) (Figure 1G). Similarly, there were no associations of NGF protein expression with that of individual pathological subtypes: compound nevi (CN, h-score = 190.7, IQR 167.5–232.1), dysplastic nevi (DN, h-score = 186.7, IQR 170.6–206.9), thin primary melanomas (TnP, h-score = 146.4, IQR 100.8–166.7), thick primary melanomas (TkP, h-score = 162.2, IQR 94.89–234.8), lymph node metastases (LNM, h-score = 109.1, IQR 62.23–276.7) and distant metastases (DM, h-score = 145.3, IQR 103.9–177.8) (Figure 1H). Gene expression analysis of *NGF* performed with GEPIA revealed a wide range of mRNA expression in both skin cutaneous melanoma (SKCM) and normal skin tissue. Observably, *NGF* in normal skin tissue was higher than SKCM, but not statistically significant (Figure 2A). *NGF* gene expression across melanoma stages was broader in range in stages 1–4 but not significantly different to stage 0 (Figure 2A). GEPIA survival data revealed that high expression of *NGF* corresponded with lower overall survival (OS) compared with low *NGF* expression (*p* = 0.049) (Figure 3A); however, there was no significant difference in disease-free survival (DFS) (Figure 3A).

### 2.2. ProNGF Expression in Melanoma

**Figure 4:**
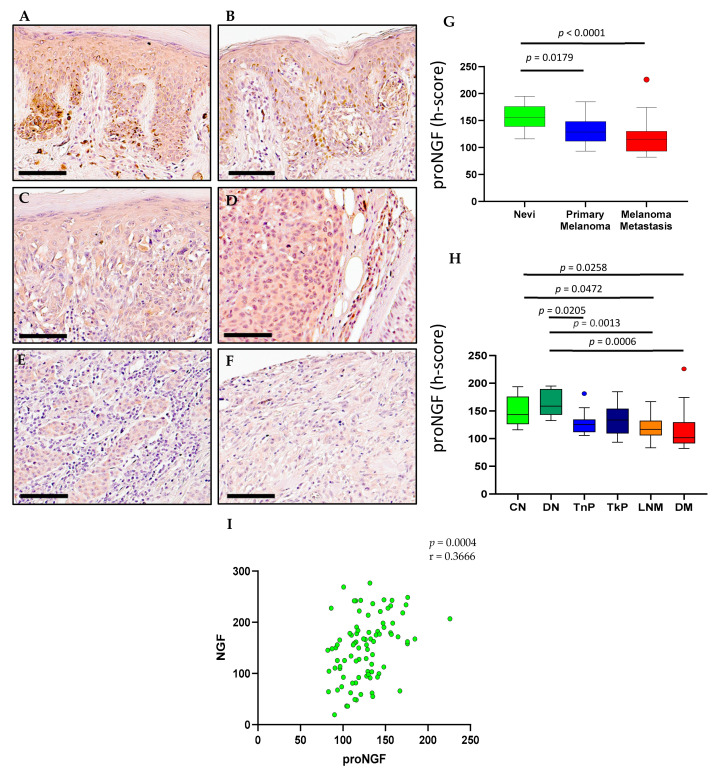
**proNGF expression in nevi, primary melanoma and metastatic human tissues** (**A**–**F**). Immunohistochemical detection of proNGF, representative pictures are shown for compound nevi (**A**), dysplastic nevi (**B**), thin primary melanomas (**C**), thick primary melanomas (**D**), lymph node metastases (**E**) and distant metastases (**F**). (**G**) Digital quantification of proNGF staining intensities according to grouped pathological subtypes: nevi (h-score = 156.2, IQR 138.9–176.1), primary melanomas (h-score = 129.0, IQR 111.8–1148.1) and metastases (h-score = 115.1, IQR 93.33–130.1). (**H**) proNGF staining intensities for individual pathological subtypes: CN = compound nevi (h-score = 143.4, IQR 126.1–176.0), DN = dysplastic nevi (h-score = 158.6, IQR 143.0–189.6), TnP = thin primary (h-score = 125.3, IQR 111.8–134.6), TkP = thick primary (h-score = 133.6, IQR 109.2–154.1), LNM = lymph node metastasis (h-score = 116.7, IQR 105.9–132.3), DM = distant metastasis (h-score = 101.8, IQR 91.64–129.7). (**I**) Correlation of proNGF and NGF stain intensities. Scale bar = 90 µm. Data are expressed as medians (horizontal line in the center of the box) and box limits indicate the interquartile range (IQR) with the whiskers extending 1.5 times the IQR from the 25th and 75th percentiles; outliers are represented by dots. H-score distributions were compared using the Wilcoxon rank-sum (dichotomous) or Kruskal–Wallis (multiple comparisons) tests (*p* < 0.05).

ProNGF immunohistochemical staining was observed in all cases of compound nevi (Figure 4A), dysplastic nevi (Figure 4B), thin primary melanomas (Figure 4C), thick primary melanomas (Figure 4D), lymph node metastases (Figure 4E) and distant metastases (Figure 4F). Digital quantification of proNGF protein expression revealed higher proNGF staining intensity in nevi (h-score = 156.2, IQR 138.9–195.0) compared to primary melanomas (h-score = 129.0, IQR 111.8–148.1, *p* = 0.0179) and metastases (h-score = 115.1, IQR 93.33–130.1, *p* < 0.0001) (Figure 4G). ProNGF staining intensities in individual pathological subtypes showed higher proNGF in compound nevi (CN, h-score = 143.4, IQR 126.1–176.0) compared to lymph node metastases (LNM, h-score = 116.7, IQR 105.9–132.3, *p* = 0.0472) and distant metastases (DM, h-score = 101.8, IQR 91.64–129.7, *p* = 0.0258) (Figure 4H). ProNGF staining intensity in dysplastic nevi (DN, h-score = 158.6, IQR 143.0189.66) was also found to be higher compared to thin primary melanomas (TnP, h-score = 125.3, IQR 111.8–134.6, *p* = 0.0205), lymph node metastasis (LNM, h-score = 116.7, IQR 105.9–132.3, *p* = 0.0013) and distant metastasis (DM, h-score = 101.8, IQR 91.64–129.7, *p* = 0.0006) (Figure 4H). Interestingly there was a positive correlation between NGF and proNGF h-scores (r = 0.3666, *p* = 0.0004) (Figure 4I). ProNGF is the precursor to NGF, therefore mRNA analysis and survival data from GEPIA were the same for NGF and had already been described (Figure 2 and Figure 3).

### 2.3. TrkA Expression in Melanoma

**Figure 5:**
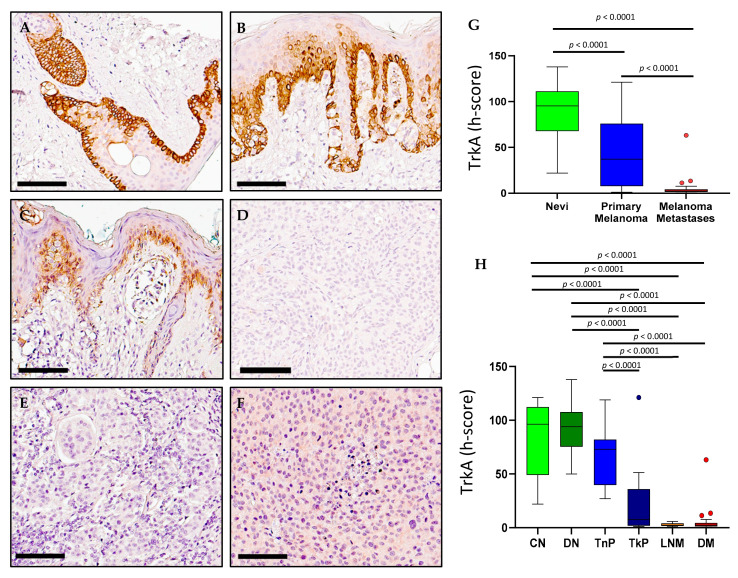
**TrkA expression in nevi**, **primary melanoma and metastatic human tissues** (**A**–**F**). Immunohistochemical detection of TrkA, representative pictures are shown for compound nevi (**A**), dysplastic nevi (**B**), thin primary melanomas (**C**), thick primary melanomas (**D**), lymph node metastases (**E**) and distant metastases (**F**). (**G**) Digital quantification of TrkA staining intensities according to grouped pathological subtypes: nevi (h-score = 95.29, IQR 67.79–111.2), primary melanomas (h-score = 37.01, IQR 7.758–76.0) and metastases (h-score = 2.421, IQR 1.491–4.261). (**H**) TrkA staining intensities for individual pathological subtypes: CN = compound nevi (h-score = 96.34, IQR 49.01–112.3), DN = dysplastic nevi (h-score = 94.00, IQR 75.32–107.7), TnP = thin primary (h-score = 72.95, IQR 39.66–82.0), TkP = thick primary (h-score = 7.730, IQR 1.854–35.90), LNM = lymph node metastasis (h-score = 2.259, IQR 1.567–4.0), DM = distant metastasis (h-score = 2.456, IQR 1.227–4.473). Scale bar = 90 µm. Data are expressed as medians (horizontal line in the center of the box) and box limits indicate the interquartile range (IQR) with the whiskers extending 1.5 times the IQR from the 25th and 75th percentiles; outliers are represented by dots. H-score distributions were compared using the Wilcoxon rank-sum (dichotomous) or Kruskal–Wallis (multiple comparisons) tests (*p* < 0.05).

TrkA immunohistochemical detection was observed to be higher in compound nevi (Figure 5A), dysplastic nevi (Figure 5B) and thin primary melanomas (Figure 5C), compared to thick primary melanomas (Figure 5D), lymph nodes metastases (Figure 5E) and distant metastases (Figure 5F). Digital quantification of TrkA immunostaining revealed higher TrkA staining intensities in the nevi tissue groups (h-score = 95.29, IQR 67.79–111.2) compared to primary melanomas (h-score = 37.01, IQR 7.758–76, *p* < 0.0001) and metastases (h-score = 2.421, IQR 1.491–4.261, *p* < 0.0001) (Figure 5G). TrkA staining intensities were higher in primary melanomas (h-score = 37.01, IQR 7.76–7.758) compared to metastases (h-score = 2.241, IQR 1.491–4.261, *p* < 0.0001) (Figure 5H). TrkA staining intensities were also higher in compound nevi (CN, h-score = 96.34, IQR 49.01–112.3), dysplastic nevi (DN, h-score = 94.0, IQR 75.32–107.7) and thin primary melanomas (TnP, h-score = 72.95, IQR 39.66–82.0) compared with thick primary melanomas (TkP, h-score = 7.730, IQR 1.854–35.90), lymph node metastases (LNM, h-score = 2.259, IQR 1.567–4.0) and distant metastases (DM, h-score = 2.456, IQR 1.227–4.473, *p* < 0.0001) (Figure 5H). Transcriptional analysis of TrkA (*NTRK1*) performed with GEPIA revealed a greater range of *NTRK1* expression log2 values in SKCM compared to normal skin tissue (Figure 2B). *NTRK1* gene expression across different stages of SKCM showed stage 5 to have the widest range of log2 values compared to stages 0–4 (*p* = 0.0187, Figure 2B). Survival statistics were retrieved from GEPIA and revealed no significant difference between high and low expression of *NTRK1* in OS and DFS (Figure 3B).

### 2.4. p75^NTR^ Expression in Melanoma

**Figure 6:**
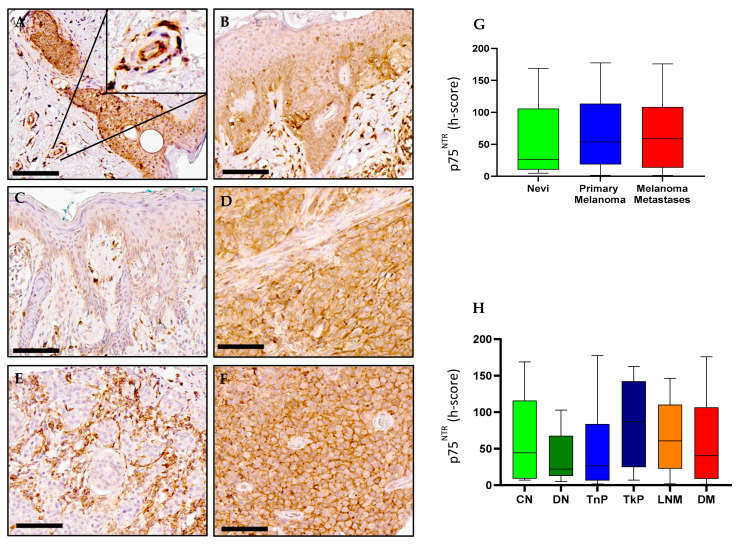
**p75^NTR^ expression in nevi**, **primary melanoma and metastatic human tissues** (**A**–**F**). Immunohistochemical detection of p75^NTR^, representative pictures are shown for compound nevi (**A**), dysplastic nevi (**B**), thin primary melanomas (**C**), thick primary melanomas (**D**), lymph node metastases (**E**) and distant metastases (**F**). (**G**) Digital quantification of p75^NTR^ staining intensities according to grouped pathological subtypes: nevi (h-score = 26.2, IQR 10.47–105.8), primary melanomas (h-score = 53.98, IQR 18.60–113.6) and metastases (h-score = 58.89, IQR 13.50–108.4). (**H**) p75^NTR^ staining intensities for individual pathological subtypes: CN = compound nevi (h-score = 44.37, IQR 8.986–116.1), DN = dysplastic nevi (h-score = 22.16, IQR 12.77–67.78), TnP = thin primary (h-score = 26.65, IQR 6.338–83.89), TkP = thick primary (h-score = 87.08, IQR 24.80–142.4), LNM = lymph node metastasis (h-score = 60.74, IQR 22.54–110.4), DM = distant metastasis (h-score = 40.46, IQR 8.702–106.8). Scale bar = 90 µm. Data are expressed as medians (horizontal line in the center of the box) and box limits indicate the interquartile range (IQR) with the whiskers extending 1.5 times the IQR from the 25th and 75th percentiles; outliers are represented by dots. H-score distributions were compared using the Wilcoxon rank-sum (dichotomous) or Kruskal–Wallis (multiple comparisons) tests (*p* < 0.05). (Enlarged insert A) p75^NTR^ staining could be observed surrounding blood vessels in some tissue samples.

P75^NTR^ immunohistochemical detection in human skin tissues revealed a wide range of staining intensities across compound nevi (CN, h-score = 44.37, IQR 8.986–116.1), dysplastic nevi (DN, h-score = 22.16, IQR 12.77–67.78), thin primary melanomas (TnP, h-score = 26.65, IQR 6.338–83.89), thick primary melanomas (TkP, h-score = 87.08, IQR 24.80–142.4), lymph node metastases (LNM, h-score = 60.74, IQR 22.54–110.4) and distant metastases (DM, h-score = 40.46, IQR 8.702–106.8); however, there were no statistical differences between each of the pathological subtypes (Figure 6A–F, respectively). Interestingly, p75^NTR^ staining could be observed surrounding blood vessels in some tissue samples (Figure 6A, enlarged insert). Transcriptional analysis of p75^NTR^ (*NGFR*) showed similar median levels were found in SKCM tissue compared to normal skin tissue (Figure 2C). Survival analysis showed no statistical difference in OS and DSF between high and low *NGFR* gene expression (Figure 3C).

### 2.5. Sortilin Expression in Melanoma

**Figure 7:**
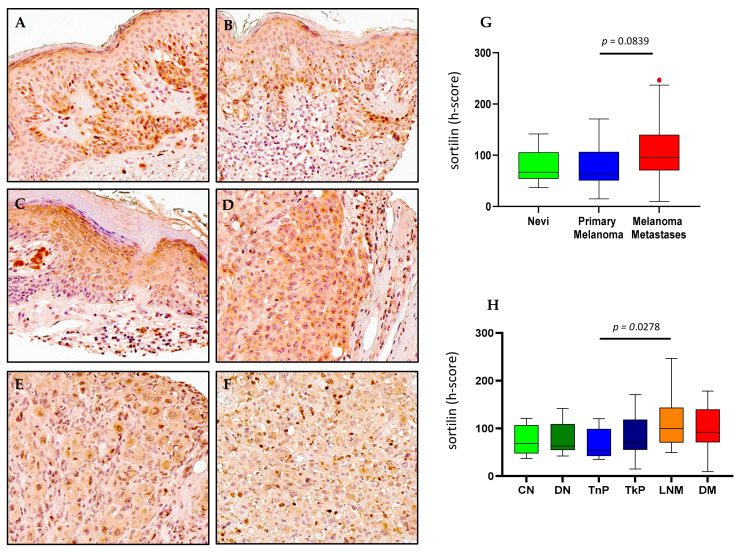
**Sortilin expression in nevi**, **primary melanoma and metastatic human tissues** (**A**–**F**). Immunohistochemical detection of sortilin, representative pictures are shown for compound nevi (**A**), dysplastic nevi (**B**), thin primary melanomas (**C**), thick primary melanomas (**D**), lymph node metastases (**E**) and distant metastases (**F**). (**G**) Digital quantification of sortilin staining intensities according to grouped pathological subtypes: nevi (h-score = 66.82, IQR 53.85–105.8), primary melanomas (h-score = 63.30, IQR 50.51–106.6) and metastases (h-score = 95.72, IQR 70.41–140.2). (**H**) Sortilin staining intensities for individual pathological subtypes: CN = compound nevi (h-score = 68.72, IQR 47.38–106.9), DN = dysplastic nevi (h-score = 62.84, IQR 54.68–109.0), TnP = thin primary (h-score = 53.92, IQR 42.20–98.84), TkP = thick primary (h-score = 70.86, IQR 55.25–118.5), LNM = lymph node metastasis (h-score = 99.67, IQR 70.11–143.7), DM = distant metastasis (h-score = 91.78, IQR 70.51–139.8). Scale bar = 90 µm. Data are expressed as medians (horizontal line in the center of the box) and box limits indicate the interquartile range (IQR) with the whiskers extending 1.5 times the IQR from the 25th and 75th percentiles; outliers are represented by dots. H-score distributions were compared using the Wilcoxon rank-sum (dichotomous) or Kruskal–Wallis (multiple comparisons) tests (*p* < 0.05).

Immunohistochemical detection of sortilin in human melanoma tissue (Figure 7A–F) revealed a broader range of stain intensities in thick primary (TkP, Figure 5D), lymph node metastases (LNM, Figure 7E) and distant metastasis (DM, Figure 7F) compared to compound nevi (CN, Figure 7A), dysplastic nevi (DN, Figure 7B) and thin primary melanomas (TnP, Figure 7C). Digital quantification of sortilin protein expression revealed no differences between grouped cases of nevi (h-score = 66.82, IQR 53.85–105.8), primary melanomas (h-score = 63.30, IQR 50.51–106.6) and metastases (h-score = 95.72, IQR 70.41–140.2) (Figure 5G), as well as the individual pathological subtypes compound nevi (CN, h-score = 68.72, IQR 47.38–106.9), dysplastic nevi (DN, h-score = 62.84, IQR 54.68–109.0) or thick primary melanomas (TkP, h-score = 70.86, IQR 55.25–118.5) (Figure 7H). Detection of sortilin in lymph node metastases (LNM, h-score = 99.67, IQR 70.11–143.7) showed higher stain intensity compared to thin primary melanomas (TnP, h-score = 53.82, IQR 42.20–98.84. *p* = 0.0278) (Figure 7H). GEPIA transcriptional analysis of sortilin (*SORT1*) revealed significantly higher expression in SKCM tissue compared to normal skin tissue (*p* < 0.01) (Figure 2D). Survival statistics appeared to show a more favorable OS outcome for patients with low expression of *SORT1*; however, this did not meet the significance cut off (*p* = 0.087) (Figure 3D). There was no difference between high and low sortilin gene expression in DFS (Figure 3D).

## 3. Discussion

In this study we clarified the expression of NGF and its receptors TrkA and p75^NTR^ in melanoma, and we reported, for the first time, the expression of proNGF and its membrane receptor, sortilin. This was the first study where all these neurotrophins and their receptors were investigated simultaneously in the same series of melanoma tumors.

Aside from their role in the development of the central and peripheral nervous system, neurotrophic growth factors are emerging as stimulators of tumor progression and metastasis [14]. In various malignancies, NGF has been reported to stimulate tumor growth and metastasis [15,16,17,18]. Targeting NGF with blocking antibodies, or inhibiting the signaling pathways with pharmacological inhibitors against its tyrosine kinase receptor TrkA, has proven to decrease tumor progression and dissemination in animal models by mechanisms involving both the direct inhibition of cancer cell growth [15,19], in addition to tumor innervation, which is essential to cancer progression [17,18]. In melanoma, recent clinical trials have shown that the pan-Trk inhibitor entrectinib is has a therapeutic impact in Trk-fusion melanoma [11], but the mechanism is uncertain as entrectinib can also inhibit other signaling molecules, such as ROS-1 or ALK.

Prior to immunohistochemical investigation, analysis of gene expression in melanoma datasets, which are available from the Cancer Genome Atlas, were performed using GEPIA. Some differences could be observed between mRNA and protein levels of TrkA. It has been reported that discrepancies between mRNA and protein expression occur in cancer [20]. Proteomic investigations have revealed that mRNA abundance does not reliably predict differences in tumoral protein abundance [21], emphasizing the importance of analyzing protein levels directly, in order to define new biomarkers and therapeutic targets in cancer. Translational regulation of TrkA or regulation of its stability has already been reported [22] and previous studies in human tumors have highlighted a similar difference between mRNA and protein levels [23].

The most striking finding of the present study was the downregulation of TrkA in melanoma primary tumors and metastases compared to nevi. Not only was TrkA expression strongly reduced in most primary melanoma, but it was also almost undetectable in metastases. This suggested that TrkA was not stimulating melanoma progression and, indeed, a previous study has shown that TrkA overexpression induced an anti-proliferative response in melanoma cell lines [10]. Therefore, loss of TrkA in melanoma could be involved in the deregulation of melanoma cell growth and future functional investigations are needed. However, the downregulation of TrkA expression in melanoma makes it unlikely that the inhibitory effect of entrectinib on melanoma progression could be due to the targeting of TrkA. The antibody that we used had not been reported to recognise TrkA fusion proteins. However, this could not be completely excluded, as our study was based solely on the use of IHC. Therefore, we needed to be careful with the interpretation, even though TrkA fusion proteins have been shown to represent less than 1% of cutaneous and mucosal melanoma [13]. In addition, ROS1 gene fusion [24] and ALK [25] are expressed and are targetable by entrectinib. Therefore, it could be hypothesized that the therapeutic effect of entrectinib was more probably attributable to targeting ROS1 or ALK; further mechanistic investigations are warranted.

The expression of proNGF and its membrane receptor sortilin, which is reported here for the first time in melanoma tumors, are of interest. Even though no significant changes in sortilin expression were observed between nevi, primary melanoma and metastases, sortilin was expressed in all melanoma and metastases samples. Specific pharmacological inhibitors have been developed against sortilin and have therapeutic potentials [26]. In other tumor types, such as breast cancer [27], thyroid [16] or pancreatic cancer [28], similar sortilin expression profiles have been described, and targeting sortilin with specific pharmacological inhibitors has been shown to strongly reduce cancer cell migration and invasion. In melanoma, the expression of sortilin was not reported but our present findings warranted the preclinical testing of sortilin inhibitors in preclinical models of melanoma, to assess their impact in tumor progression and dissemination.

## 4. Materials and Methods

### 4.1. Skin and Melanoma Tissue Samples

High-density tissue microarrays (TMA) were constructed from formalin-fixed paraffin-embedded (FFPE) melanocytic tumor tissues retrieved from the Department of Tissue Pathology and Diagnostic Oncology at the Royal Prince Alfred Hospital, Australia. Tissue samples totaled 100 cases and were made up of the following subtypes: compound nevi (*n* = 10), dysplastic nevi (*n* = 10), thin primary melanoma (<1 mm Breslow depth, *n* = 20), thick primary melanoma (>1 mm Breslow depth, *n* = 20), lymph node metastases (*n* = 20) and distant metastases (*n* = 20). Tissues were individually examined by a pathologist and certified according to WHO published standardizations of diagnosis, classification and pathological grade. Studies were approved by the Human Research Ethics Committee of The University of Newcastle, Australia (X11-0023 and H-2012-0063) and Royal Prince Alfred Hospital, Australia (HREC/11/RPA) and were in agreement with the guidelines set forth by the Declaration of Helsinki. All participants provided written informed consent.

### 4.2. Immunohistochemistry

Immunohistochemistry (IHC) was performed as previously described [29]. After deparaffinization and rehydration of TMA slides following standard procedures, heat induced epitope retrieval was carried out in a low pH, citrate-based antigen unmasking solution (catalogue number H-3300, Vector Laboratories, Burlingame, CA, USA) using a decloaking chamber (catalogue number DC2002, Biocare Medical, Pacheco, CA, USA) at 95 °C for 20 min and 90 °C for 10 s. IHC was then performed using an ImPRESS Horse Anti-Rabbit IgG Polymer Detection Kit (Peroxidase) (Catalogue number MP-7401, Vector Laboratories, Burlingame, CA, USA), as per the manufacturer’s recommendations. Briefly, endogenous peroxidases were inactivated with 0.3% H_2_O_2_ (catalogue number HA154, Chem-Supply Pty Ltd., Gillman, SA, Australia) and blocked with 2.5% horse serum. The following primary antibodies were applied: anti-proNGF (0.8 µg/mL, catalogue number ANT005, Alomone labs, Jerusalem, Israel), anti-NGF (13.3 µg/mL, catalogue number ab52918, Abcam, VIC, Australia), anti-sortilin (0.8 µg/mL, catalogue number ANT009, Alomone labs, Jerusalem, Israel), anti-p75^NTR^ (2 µg/mL, catalogue number ANT007, Alomone labs, Jerusalem, Israel), anti-TrkA (1:200 dilution, catalogue number cs-2508, Cell Signaling Technology, Danvers, MA, USA). Immunohistochemical negative control testing was also performed using the following antibodies diluted to match highest primary antibody concentrations: rabbit IgG, purified serum nonimmune, isotype control (catalogue number 20009, Alpha Diagnostic International, San Antonio, TX, USA) or rabbit (DA1E) monoclonal antibody IgG Isotype Control (catalogue number 3900, Cell Signaling Technology), and are shown in Supplementary Materials Figure S1. Secondary antibodies (catalogue number MP-7401, Vector Laboratories, Burlingame, CA, USA) were applied to the sections and revealed with DAB Peroxidase (HRP) Substrate Kit (catalogue number SK-4100, Vector Laboratories, Burlingame, CA, USA). Finally, TMA slides were counterstained with Hematoxylin QS (catalogue number LS-J1045, Vector Laboratories, Burlingame, CA, USA), dehydrated and cleared in xylene before mounting in Ultramount No.4 (catalogue number UM5-T, Hurst Scientific, Forrestdale, WA, Australia).

### 4.3. Digital Quantification of Immunohistochemistry

Following IHC staining, TMAs were digitized using the Aperio AT2 scanner (Leica Biosystems, Mount Waverley, VIC, Australia) at 40× absolute resolution. Quantitative IHC analyses were performed using the HALO™ image analysis platform (version 2.3, Indica Labs, Albuquerque, NM, USA). Tissue classification algorithms were used to differentiate tissues, and pixel intensity values corresponding to DAB staining were calculated using the area quantification algorithm [30], which detects and quantifies protein expression in the membrane and cytoplasm. Pixel intensity values were then used to determine the h-scores for each core (index calculated as the sum of 3× % of pixels with strong staining, +2× % pixels with intermediate staining, +1× % pixels with weak staining). Area quantification does not differentiate between cell compartments and so the h-score was an overall score of stain intensity of the area. Tumor and areas of nevi were selected for analysis while the stroma and surrounding tissue was excluded from the analysis. Importantly, DAB staining was differentiated from regions containing melanin pigmentation by visually assessing each of the TMAs with a trained pathologist, before and after IHC was performed, and manually excluding regions with high levels of melanin pigmentation during digital quantification. H-scores were analyzed as continuous variables, with summary statistics presented as group level medians and interquartile ranges (IQR). H-score distributions were compared using the Wilcoxon rank-sum (dichotomous) or Kruskal–Wallis (multiple comparisons) tests. To assess the primary hypothesis (difference in neurotrophin and receptor expression between pathological subtypes), a two-sided alpha of 0.05 was used. Statistical analyses were based on complete cases and performed using Prism (version 8.2.0, GraphPad Software, San Diego, CA, USA).

### 4.4. GEPIA Database

Gene Expression Profiling Interactive Analysis (GEPIA) (http://gepia.cancer-pku.cn) (accessed on 26 July 2021) is a recently developed interactive web application for gene expression analysis based on RNA sequencing and expression of 9736 tumors and 8587 normal samples from the Cancer Genome Atlas (TCGA) [31] and Genotype-Tissue Expression (GTEx) [32] databases, using a standard processing pipeline [33]. In this study, mRNA expressions between skin cutaneous melanoma (SKCM) and normal skin tissue, as well as different stages of melanoma, were compared with GEPIA, and a survival analysis in SKCM comparing high and low gene expression of *NGF*, *NTRK1*, *NGFR* and *SORT1* was also conducted. GEPIA uses one-way ANOVA for differential analysis of gene expression, using disease states (SKCM or normal) as variables for the box plots and pathological stages (stage 0-IV) as variables for the stage plots. GEPIA uses the log-rank test for overall survival and disease-free survival analysis.

## 5. Conclusions

In conclusion, exploratory pathological analyses, such as the one we have performed here, are useful ways not only to identify new biomarkers of cancer, but also to highlight potential therapeutic targets. Overall, this study pointed to the absence of TrkA expression as a potential biomarker of melanoma progression and to sortilin as a potential new therapeutic target. Further preclinical and clinical studies are warranted to test these hypotheses.

## Data Availability

The data presented in this study are available on request from the corresponding author.

## Supplementary Materials

The following supporting information can be downloaded at: https://www.mdpi.com/article/10.3390/ijms23084260/s1.
