# Supplementary material for: Expression of NGF/proNGF and Their Receptors TrkA, p75NTR and Sortilin in Melanoma
Source: Int J Mol Sci. 2022 Apr 12;23(8):4260. doi: 10.3390/ijms23084260 (PMC9032112; doi:10.3390/ijms23084260)
Supplement: Supplementary file 1 [file ijms-23-04260-s001.zip › ijms-1681150-supplementary.pdf]

Supplementary Data

Expression of NGF/proNGF and their receptors TrkA, $p75^{NTR}$ and sortilin in melanoma

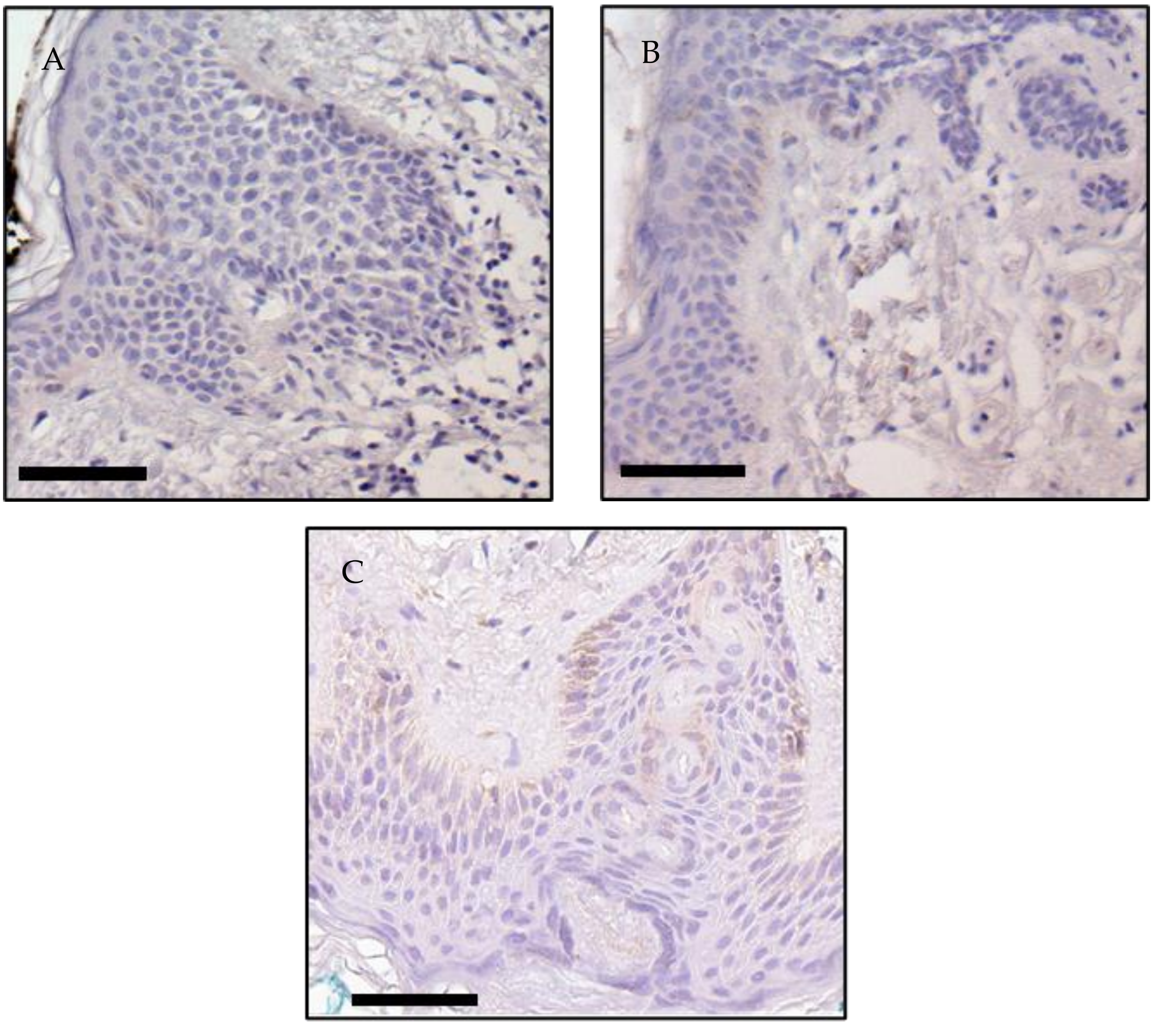

Supplementary Figure S1. Isotype negative controls for immunohistochemistry. (A) Immunostaining was performed without addition of any primary antibody. (B) Rabbit (DA1E) mAb IgG Isotype control (diluted to match highest primary antibody concentration; catalogue number 3900S, Cell Signaling Technology, Danvers, MA, USA). (C) Rabbit IgG, purified serum nonimmune, isotype control (diluted to match highest primary antibody concentration; catalogue number 20009, Alpha Diagnostic International). Scale bar = 90μm.
